# Autoimmune obsessive-compulsive disorder with novel anti-CNS autoantibodies in cerebrospinal fluid

**DOI:** 10.1038/s41380-022-01688-3

**Published:** 2022-07-13

**Authors:** Dominique Endres, Benjamin Pankratz, Tilman Robinson, Karoline Pitsch, Theresa Göbel, Kimon Runge, Andrea Schlump, Kathrin Nickel, Marco Reisert, Horst Urbach, Ulrich Voderholzer, Nils Venhoff, Katharina Domschke, Harald Prüss, Miriam A. Schiele, Ludger Tebartz van Elst

**Affiliations:** 1 Department of Psychiatry and Psychotherapy, Medical Center—University of Freiburg, Faculty of Medicine, University of Freiburg, Freiburg, Germany; 2 Department for Neurology, Medical Center—University of Freiburg, Faculty of Medicine, University of Freiburg, Freiburg, Germany; 3 Department of Diagnostic and Interventional Radiology, Medical Physics, Medical Center—University of Freiburg, Faculty of Medicine, University of Freiburg, Freiburg, Germany; 4 Department of Stereotactic and Functional Neurosurgery, Medical Center—University of Freiburg, Faculty of Medicine, University of Freiburg, Freiburg, Germany; 5 Department of Neuroradiology, Medical Center—University of Freiburg, Faculty of Medicine, University of Freiburg, Freiburg, Germany; 6 Schoen Clinic Roseneck, Prien am Chiemsee, Germany; 7 Department of Psychiatry and Psychotherapy, University Hospital Munich, Munich, Germany; 8 Department of Rheumatology and Clinical Immunology, Medical Center—University of Freiburg, Faculty of Medicine, University of Freiburg, Freiburg, Germany; 9 Center for Basics in Neuromodulation, Medical Center—University of Freiburg, Faculty of Medicine, University of Freiburg, Freiburg, Germany; 10 Department of Neurology and Experimental Neurology, Charité—Universitätsmedizin Berlin, Berlin, Germany; 11 German Center for Neurodegenerative Diseases (DZNE), Berlin, Germany

**Keywords:** Diagnostic markers, Neuroscience

## To the Editor:

In a recent review article, Howes et al. summarized the current state of treatment resistance in psychiatry including obsessive-compulsive disorder (OCD) [1]. However, while pseudotherapy resistance may also be caused by unrecognized autoimmune encephalitis, it still plays a minor role in psychiatry [2, 3]. Previous such psychiatric autoimmune encephalitis cases mostly presented as autoimmune psychoses [3]. Particularly, little is known about “autoimmune OCD” as a potential nosological entity in adulthood [4]. Therefore, this article presents a paradigmatic patient with OCD symptoms that are most likely autoimmune in origin and associated with novel anti-central nervous system (CNS) autoantibodies and responsiveness to immunotherapy.

**Fig. 1:**
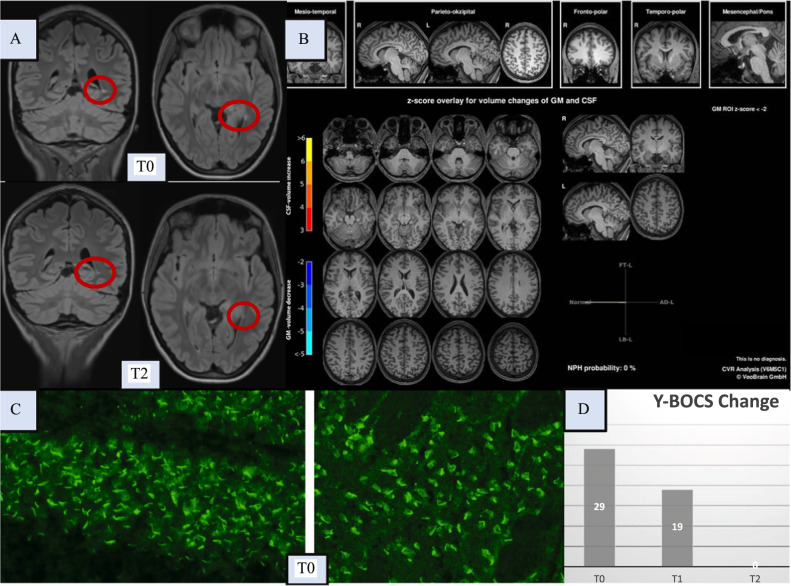
Diagnostic findings initially (at T0) and after immunotherapy (at T1/T2). Here, the magnetic resonance imaging (MRI) findings of the brain, the autoantibody findings, and the clinical course based on the Yale-Brown Obsessive-Compulsive Scale (Y-BOCS) scores are shown. Brain [^18^F]fluorodeoxyglucose positron emission tomography (FDG-PET) identified unremarkable findings, and no evidence of malignancy was identified in the whole body FDG-PET. Optical coherence tomography and electroencephalography were normal (these normal findings are not shown). **A** The left upper row shows the conspicuous FLAIR MRI findings of the brain with a solitary lesion in the left tapetum (at T0), atypical for age in location and localization and potentially (post)inflammatory, but the MRI criteria for multiple sclerosis were not met. In addition, a pineal gland cyst was found loco typico without a space-occupying effect (max. 11 mm in diameter; not shown in detail). The lower images show a regressive lesion in the left tapetum after clinical full remission (at T2, approximately 7 weeks after steroid pulse treatment). **B** A combined volume- and region-based analysis method (CVR; https://www.veobrain.com/?page=veomorph) detected no atrophy at T0. At time point T2, there was no relevant change (not shown). **C** In the cerebrospinal fluid (CSF), autoantibodies against granule cell cilia were found in the hippocampus (left in the bottom row; “rings and rods” pattern) and in neurons of the cortex (right in the bottom row; at T0). In addition, several large vessels were stained (not shown). The tissue-based assay using serum material revealed non-specific findings (not shown). The well-characterized anti-central nervous system autoantibodies against intracellular antigens (Yo/Hu/CV2/CRMP5/Ri/Ma1/2/SOX1/Tr/Zic4/GAD65/amphiphysin) or glial structures (AQP4/MOG) in serum and against cell surface antigens (NMDA-R/LGI1/CASPR2/AMPA1/2-R/GABA-B-R/DPPX) in serum and CSF remained unremarkable (not shown). Additional CSF analyses identified pleocytosis, with a white blood cell count of 10/µl (reference <5/µl); intrathecal immunoglobulin (Ig) synthesis of two isotypes, IgG (53%) and IgA (48%; reference both <10%); an elevated IgG index of 1.37 (reference <0.7); and CSF-specific oligoclonal bands. In contrast, the albumin quotient was normal, and pathogen testing of the CSF remained unremarkable. Elevated specific antibody indices (AIs) for HSV (2.3; reference <1.5) and VZV (2.8; reference <1.5) suggested polyclonal IgG synthesis, but the MRZ reaction remained negative (i.e., AIs for measles and rubella were negative). **D** The patient showed an initial Y-BOCS score of 29 points (at T0). At T1, immediately after steroid pulse treatment, the patient was able to better distance herself from the obsessive-compulsive symptoms (OCS), and the Y-BOCS score decreased to 19 points. Full remission of OCS (Y-BOCS score: 0) was evident at T2 (approximately 7 weeks after steroid pulse treatment). Neuropsychological testing of attentional performances revealed an improvement in reaction time (alertness with/without warning tone) at the time of the second assessment (directly after stopping steroids; not shown).

The female student, in her mid-20s, gradually developed severe obsessive-compulsive symptoms (OCS) over ~1 year during the COVID-19 pandemic, with washing compulsions and fear of contamination, initially related to SARS-CoV-2 and subsequently to other pathogens. She felt the urge to wash her hands at least 20–30 times a day in a ritualized manner for prolonged periods and avoided touching many objects (e.g., door handles) for fear of contamination. In addition, control compulsions emerged (e.g., frequent checking of electrical appliances). Ego dystonia of these symptoms was maintained throughout, and no psychotic symptoms occurred. The OCS were accompanied by moderate depressiveness. As the patient no longer felt able to leave her house, treatment in an outpatient setting was ruled out; therefore, she was admitted as an inpatient. The clinical diagnosis of OCD was confirmed by the Structured Clinical Interview for DSM-IV. The Yale-Brown Obsessive-Compulsive Scale (Y-BOCS) assessment yielded a score of 29 points at admission (T0; 24–31 “severe” symptoms). A hypochondriac personality structure had been described previously; hence, a psychoreactive origin of OCS triggered by the COVID-19 pandemic seemed likely. The neurological examination remained unremarkable. A somatic baseline workup revealed the presence of *antinuclear autoantibodies in serum with a homogeneous nuclear immunofluorescence pattern on HEp2 cells and a weak (+) specificity against proliferating cell nuclear antigen in extractable nuclear antigen testing*. However, the further workup did not support a diagnosis of systemic lupus erythematosus. Magnetic resonance imaging (MRI) of the brain revealed *a single periventricular lesion in the left lower horn (“tapetum”)* without contrast uptake. In light of the probably (post)inflammatory MRI lesion, a cerebrospinal fluid (CSF) analysis was performed, *which revealed inflammatory CSF signals with slight pleocytosis (10/µl; reference <*5*/µl), intrathecal immunoglobulin (Ig) synthesis of two isotypes (IgG and IgA), an elevated IgG index, and CSF-specific oligoclonal bands.* Streptococcal antibodies and serologies for hepatitis B and C were negative. The well-characterized anti-CNS autoantibodies remained unremarkable; however, *tissue testing from CSF with indirect immunofluorescence on unfixed mouse brain sections according to an established protocol* [5] *showed an IgG autoantibody binding pattern, especially against the cilia of granule cells in the hippocampus, but also in the cortex, as well as against several large vessels (“rings and rods” pattern)*. Anti-inosine-5′-monophophate dehydrogenase (IMPDH) antibodies—which can show a similar pattern—were negative [6]. Because of probable autoimmune etiology, immunotherapy was initiated with the patient’s written informed consent. Thereafter, steroid pulse treatment with 500 mg/day methylprednisolone intravenously over 5 days followed by oral methylprednisolone treatment (starting with 40 mg/day) and stepwise tapering over 30 days was initiated. In the week following the steroid pulse (at T1), the patient was able to better distance herself from OCS, and the Y-BOCS score dropped to 19. Subsequently, the patient received supervised exposure therapy (after previous preparation during the diagnostic period). Approximately 7 weeks (at T2) after starting immunotherapy with steroids and after psychotherapy, no OCS remained (Y-BOCS score of 0 points). The patient retrospectively reported not having felt any anxiety during the exposure exercises. Following exposure treatment, she was able to “unlearn” the avoidance behavior without any problems. The MRI lesion in the left tapetum clearly regressed (Fig. 1). Relapse prophylaxis with azathioprine was suggested.

The case is paradigmatic, demonstrating a “classical OCD” manifestation that initially appeared psychoreactive, with inflammatory MRI and CSF findings and a novel anti-CNS autoantibody pattern in the CSF.

In the literature so far, OCS have been described only rarely associated with well-characterized autoantibodies [4]. However, the current case study shows that the spectrum of autoantibodies against CNS targets in patients with OCS may be broader than previously known.

From a pathophysiological perspective, autoantibodies against the cilia of granule cells in the hippocampus were detected. Granule cells can elicit excitatory effects via glutamate in the hippocampus and cerebellum and inhibitory effects via GABAergic mechanisms in the olfactory bulb [7]. Glutamatergic dysregulation is increasingly discussed in the context of OCD; therefore, autoantibodies against granule cells may be another jigsaw piece in the context of the “glutamate theory of OCD” [8]. In addition, large vessels were stained. Similar binding patterns have been described in appearance as “rings and rods” and are reminiscent of anti-IMPDH antibodies, which are directed against subcellular filaments composed of the enzyme inosine-5′-monophophate dehydrogenase [6]. Anti-IMDPH antibodies were reported to be associated with interferon and ribavirin therapy in hepatitis C [6]. However, anti-IMPDH antibodies and serology for hepatitis C were negative in this patient, who had never been treated with interferon or ribavirin. Autoantibodies against vessels might also disturb blood–CSF barrier function and therefore promote CNS autoimmunity [9].

From a clinical viewpoint, the course of the disease is striking. The patient was able to rapidly “unlearn” the OCS and eliminate avoidance behavior, while no “anxiety-compulsion dynamics” emerged during the parallel psychotherapy exposures. We hypothesize that this increased distance from the OCS was caused by the anti-inflammatory effect of the immunotherapy. Furthermore, improved brain-imaging results, with the MRI lesion receding in the tapetum; the strong reduction of the Y-BOCS score from 29 to 0; and the temporal association with the steroid treatment support the presence of autoimmune OCD [4].

*In summary*, in some cases, classical OCD symptoms may have an underlying autoimmune cause. Considering that treatment resistance rates in OCD are high [1], a further workup of such cases would be clinically relevant, as this may provide personalized treatment alternatives for a small subgroup of patients with autoimmune OCD and prevent therapy resistance.

## Data Availability

All necessary data can be found in the paper.

## References

[1] Howes OD, Thase ME, Pillinger T. Treatment resistance in psychiatry: state of the art and new directions. Mol Psychiatry. 2021. 10.1038/s41380-021-01200-3. 10.1038/s41380-021-01200-3 PMC8960394 34257409

[2] Prüss H (2021). Autoantibodies in neurological disease. Nat Rev Immunol.

[3] Endres D, Lüngen E, Hasan A, Kluge M, Fröhlich S, Lewerenz J, et al. Clinical manifestations and immunomodulatory treatment experiences in psychiatric patients with suspected autoimmune encephalitis: a case series of 91 patients from Germany. Mol Psychiatry. 2022. 10.1038/s41380-021-01396-4. 10.1038/s41380-021-01396-4 PMC9095476 35046526

[4] Endres D, Pollak TA, Bechter K, Denzel D, Pitsch K, Nickel K (2022). Immunological causes of obsessive-compulsive disorder: is it time for the concept of an “autoimmune OCD” subtype?. Transl Psychiatry.

[5] Kreye J, Reincke SM, Kornau HC, Sánchez-Sendin E, Corman VM, Liu H (2020). A therapeutic non-self-reactive SARS-CoV-2 antibody protects from lung pathology in a COVID-19 Hamster Model. Cell..

[6] Calise SJ, Chan EKL (2020). Anti-rods/rings autoantibody and IMPDH filaments: an update after fifteen years of discovery. Autoimmun Rev..

[7] Chadderton P, Margrie TW, Häusser M (2004). Integration of quanta in cerebellar granule cells during sensory processing. Nature.

[8] Pittenger C, Bloch MH, Williams K (2011). Glutamate abnormalities in obsessive compulsive disorder: neurobiology, pathophysiology, and treatment. Pharmacol Ther.

[9] Shimizu F, Schaller KL, Owens GP, Cotleur AC, Kellner D, Takeshita Y (2017). Glucose-regulated protein 78 autoantibody associates with blood-brain barrier disruption in neuromyelitis optica. Sci Transl Med.

